# Conservation of the pure adiabatic state in Ehrenfest dynamics of the photoisomerization of molecules

**DOI:** 10.1038/srep18220

**Published:** 2015-12-11

**Authors:** Yoshiyuki Miyamoto, Yoshitaka Tateyama, Norihisa Oyama, Takahisa Ohno

**Affiliations:** 1 Research Center for Computational Design of Advanced Functional Materials, National Institute of Advanced Industrial Science and Technology (AIST), Central 2, 1-1-1 Umezono, Tsukuba, Ibaraki 305–8568, Japan; 2 International Center for Materials Nanoarchitectonics (MANA), National Institute for Materials Science (NIMS), 1-1 Namiki, Tsukuba, Ibaraki 305-0044, Japan; 3 Computational Material Science Unit, National Institute for Materials Science (NIMS), 1-2-1 Sengen, Tsukuba, Ibaraki 305–0047, Japan

## Abstract

We examined real-time-propagation time-dependent density functional theory (rtp-TDDFT) coupled with molecular dynamics (MD), which uses single-particle representation of time-evolving wavefunctions allowing exchange of orbital characteristics between occupied and empty states making the effective Kohn-Sham Hamiltonian dependent on the potential energy surfaces (PESs). This scheme is expected to lead to mean-field average of adiabatic potential energy surfaces (PESs), and is one of Ehrenfest (mean-field) approaches. However, we demonstrate that the mean-field average can be absent in simulating photoisomerization of azobenzene and ethylene molecules. A transition from the S2 to the S1 excited state without the mean- field average was observed after examining several rtp-TDDFT-MD trajectories of a photoexcited azobenzene molecule. The subsequent *trans-cis* isomerization was observed in our simulation, which is consistent with experimental observation and supported by previous calculations. The absence of the mean-field average of PESs was also observed for the transition between the S1 and S0 states, indicating that the MD simulation was on a single PES. Conversely, we found no transition to the ground state (S0 state) when we performed a MD simulation of an S1 excited ethylene molecule owing to the constraint on the occupation number of each molecular orbital. Thus, we conclude that, at least for azobenzene and ethylene molecules, the rtp-TDDFT-MD is an on-the-fly simulation that can automatically see the transition among the PESs of excited states without the mean-field average unless the simulation reaches the PES of the S0 state.

MD simulations in electronic excited states are useful for photoinduced dynamics. A major problem in excited-state dynamics in materials is the transition among adiabatic potential energy surfaces (PESs). Based on the many-body wavefunction theory of quantum chemistry, the surface hopping approach^1^,^2^,^3^ using multiple MD trajectories allowed excited-state dynamics to be studied by expressing the time-dependent electron wavefunctions as a linear combination of adiabatic wavefunctions with time-dependent coefficients. Because the Hamiltonian is common to molecular orbitals (MOs) no matter which PES the set of MOs belongs to, the transition matrix element can be computed directly. However, this approach becomes practically difficult as the size of molecules increases. This is because of the increase in the Slater-determinant size, the number of PESs involved in the transition, and the number of MD trajectories necessary for surface hopping operations, even when a single initial atomic configuration is used.

An alternative approach to the many-body wavefunction theory of quantum chemistry is density functional theory (DFT)^4^,^5^ using the single-particle representation for wavefunctions of electrons with a Hamiltonian depending on the charge density of electrons. Combining surface-hopping with DFT is valid within linear-response time-dependent DFT (LR-TDDFT) that only considers the charge density of the electron ground state^6^,^7^,^8^,^9^. When the charge density depends strongly on PESs, the Kohn-Sham Hamiltonian should deviate from that of the ground state, meantime, the LR-TDDFT can follow the change of the self-consistent potential within linear response to the change of the charge^10^.

On the other hand, an Ehrenfest approach^11^ combined with real-time electron propagation by solving the time-dependent Schrödinger equation allows us to perform unbiased on-the-fly simulations of excited-state MD. The transition among PESs is approximated as a mean-field average of several PESs, as discussed in ref. 2. Because real-time propagation time-dependent density functional theory (rtp-TDDFT)^12^,^13^ coupled with MD is an Ehrenfest approach, the mean-field average of PESs is expected to arise. If so, the MD simulation is performed under the *mean* forces, and multiple MD trajectories are required.

Suppose we start from the singlet S_n_ excited state of a molecule and experience the transition toward a singlet S_n−1_ excited state. This transition is allowed without changing the set of occupation numbers of MOs by alternating the order of the energy levels of differently occupied MOs. Figure 1(a) shows this scenario with a schematic of a single-particle transition between two MOs. If the S_n_ → S_n−1_ transition occurs, rtp-TDDFT-MD should show the potential energy curve from the S_n_ to the S_n−1_ state (Fig. 1(b)). Otherwise, the rtp-TDDFT-MD shows the mean-field average of the S_n_ and S_n−1_ states by taking the intermediate value of the potential (Fig. 1(c)).

In this paper, we examined the rtp-TDDFT-MD approach to photoexcited molecular systems to determine which of the scenarios in Fig. 1(b,c) occurs. We chose practical, common examples. The first case is photoinduced S_2_ excitation and transition to the S_1_ state in azobenzene and subsequent *trans-cis* isomerization. The other case is an S_1_ excited ethylene (C_2_H_4_) molecule for examining the S_1_ to S_0_ transition. For both cases, we did not see the mean-field average shown in Fig. 1(b). Azobenzene undergoes a smooth S_2_ → S_1_ transition, whereas C_2_H_4_ shows no S_1_ → S_0_ transition. Thus, we conclude that, at least for the current cases, the rtp-TDDFT-MD is an on-the-fly tool to model the PES transition automatically unless the simulation reaches the PES of the S_0_ state. (Note that the current scheme did not show the decay into the ground state.).

## Computational schemes

To examine the presence or absence of the mean-field average of PESs in the rtp-TDDFT-MD simulation, we used the local density approximation (LDA)^14^ for the adiabatic exchange-correlation functional of DFT. The LDA was used because there are previous studies of azobenzene^15^ that show that PESs for the S_0_, S_1_, and S_2_ states obtained with several types of DFT exchange-correlation functional, such as the BLYP^16^,^17^ and PBE functional^18^ of the generalized gradient approximation and LDA, are similar to each other, and to those obtained by the many-body wavefunction theory of quantum chemistry with a higher level of accuracy including the complete active space self-consistent field (CASSCF) method^19^. LDA also performed well for the photoinduced reaction of diazomethane, which is a precursor of carbene^20^ and the S_1_ excited-state MD of C_2_H_4_ presented in this work, which is consistent with ref. 21.

The calculation was performed by using plane-wave total energy formalisms^22^ and the norm-conserving pseudopotentials^23^. The cutoff energy for the plane-wave basis set was set as 60 Ry. The rtp-TDDT-MD simulation was performed by using the code developed in ref. 24. The real-time propagation of the electron wavefunctions by solving the time-dependent Kohn-Sham equation,


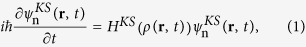


was computed by applying the Suzuki-Trotter split operator method^25^,^26^ with a time-step interval of 0.03 a.u. (1 a.u. = 2.42 × 10^−2^ fs). Here, 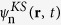 is the *n*-th time-dependent Kohn-Sham orbital and 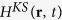 is the Kohn-Sham Hamiltonian of TDDFT^12^. 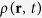 is the electron charge density consisting of the norm of all occupied Kohn-Sham orbitals. To perform the MD simulations, forces were derived from the same formalism as the Hellmann-Feynman force for the plane wave scheme^22^. To prepare the excited state, we manually set occupation numbers of some MOs as half and performed the self-consistent calculation as in ref. 27 within the constraint DFT (ΔSCF) scheme. The validation of using the ΔSCF scheme has been discussed thoroughly and is summarized in ref. 28, and agreement with PES by TDDFT was reported for azobenzene in ref. 15. We employed a spin-unpolarized approximation, assuming that half-occupied MOs consist of 0.5 electrons with an up spin and 0.5 electrons with a down spin.

## Results for azobenzene

### Optical and orbital properties

First, we optimized the molecular structure of azobenzene in the electronic ground state. The cell size was 20 × 14 × 14 Å^3^ with the longest lattice vector parallel to the long molecular axis of azobenzene. We assumed no symmetric restriction in determining the atomic coordinates and the electron charge density of azobenzene. Atomic coordinates include an error of ±0.042 Å that was derived from the averaged deviation in equivalent atomic pairs of an azobenzene molecule after *C*_2_ rotation around the center of the N = N bond. The optimized atomic coordinates are listed in Table S1 in the supplementary material. According to the optical matrix elements obtained by using the Kohn-Sham orbital from *static* LDA calculations, the magnitude of the oscillator strength for the S_2_ excitation is stronger than the S_1_ excitation. The corresponding excitation energies for the S_1_ and S_2_ excitations are 2.48 and 3.37 eV, respectively, obtained by a Fourier transformation of the dipole dynamics induced by a short pulse^29^,^30^ based on rtp-TDDFT. These quantities are comparable to experimental data^31^ with an error of ~0.3 eV for S_1_ and ~0.7 eV for S_2_.

The HOMO-1 and LUMO orbitals individually have N-N *π* and π* characters, as shown by the contour maps of the norm of each MO in Fig. 2. These orbital characters obtained by LDA are consistent with theoretical natural orbital plots that were obtained by the CASSCF method^32^.

### rtp-TDDFT-MD electron-ion dynamics

The rtp-TDDFT-MD simulations of azobenzene were performed with no symmetry constraints. At the beginning of the rtp-TDDFT-MD simulation, all ions had no initial velocities. We used four initial conditions, one of which was the optimized atomic geometry and others were obtained by sampling the atomic geometries of snapshots of a single trajectory of the ground-state MD simulation. The initial condition of the MD simulation was assumed with randomized ionic velocities with a corresponding temperature of 230 K. The four initial atomic coordinates are listed in Tables S1 to S4 in the supplementary materials with a potential energy variation of up to 0.5 eV per azobenzene molecule.

We compared the potential energy of the rtp-TDDFT-MD simulation


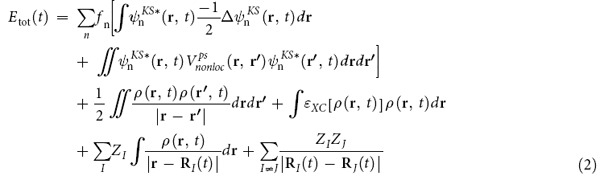


with the potential energy obtained by *static* DFT, which is also expressed by equation (2), but without *t* dependence. In these four rtp-TDDFT-MD trajectories, sets of coordinates obtained from several snapshots were used to calculate the potential by *static* DFT for the ground state, and S_1_ and S_2_ excited states within the ΔSCF scheme. In equation (2), 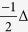 is the kinetic-energy operator. 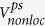 is the sum of the non-local terms of the pseudopotentials. 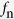 is the occupation number, which was manually set for excited states. 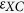 is the exchange-correlation potential as a functional of the charge density. 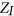 and 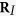 are the charge and coordinate of the *I*-th ion, respectively.

By setting the S_2_ excited state as an initial condition, the rtp-TDDFT simulations were continued to 11 fs, which induced the S_2_ → S_1_ transition, as described later. Figure 3 shows the results for the time-evolution of the rtp-TDDFT potential energy of the four trajectories, in which 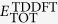 is the potential energy obtained by equation (2) throughout the rtp-TDDFT-MD simulations. Solid squares, circles, and triangles show the potential energies of S_0_, S_1_, and S_2_ states obtained by the *static* DFT at the atomic coordinates for the corresponding times determined by the rtp-TDDFT-MD simulations. For all trajectories, the potential energy obtained by the rtp-TDDFT-MD simulation followed the PES of the S_2_ state at the beginning then switched to that of the S_1_ state after 6 fs, which indicates that the lifetime of the S_2_ state is short. (One can note that the 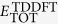 is in midpoint between S_2_ and S_1_ state from 4 fs to 6 fs in “Trajectory 1” of Fig. 3 but follows S_1_ state later than 6 fs. In such a short time-constant, the influence on ionic motion is negligibly small, thus MD simulation will follow a trajectory of S_1_ state later than 6 fs).

**Figure 1:**
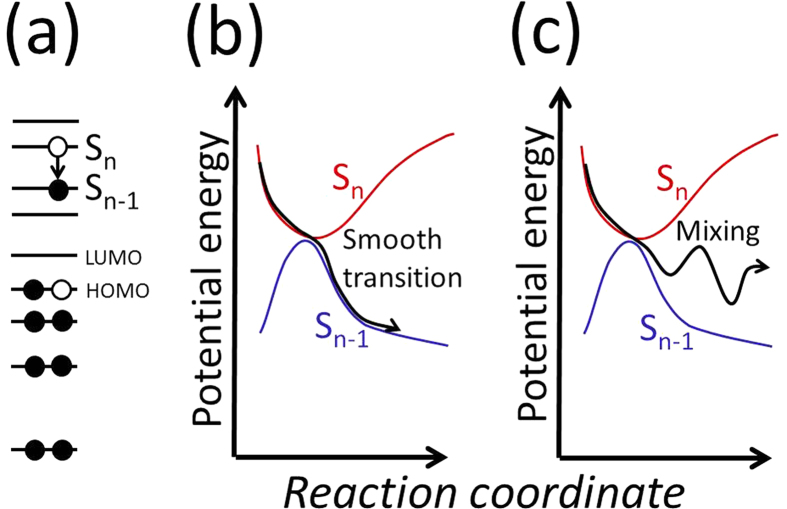
Schematics of the S_n_ → S_n−1_transition. (**a**) Single-particle transition from the MO of the S_n_ excited state to that of the S_n−1_ excited state. (**b**) Transition and PES of the S_n_ state to the PES of the S_n−1_ state, and (**c**) Mean-field average of the S_n_ and S_n−1_ states throughout the simulation.

**Figure 2:**
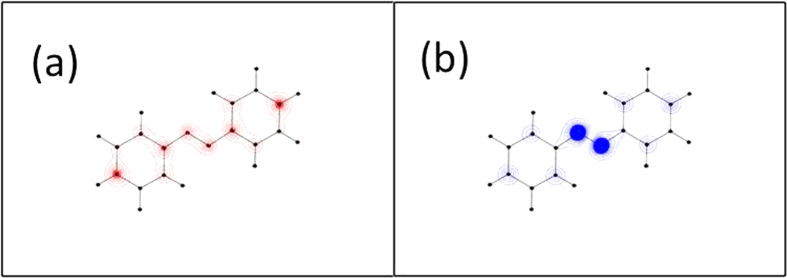
Contour maps of norms of (a) HOMO-1 and (b) LUMO states of an azobenzene molecule. The contour line interval is set as 0.019 *e*/Å^3^, which is also a common minimum value of the contour lines in (**a**,**b**).

Even at the transition from the S_2_ to the S_1_ states, the original orbital characters of HOMO-1 and LUMO remained as same as those displayed in Fig. 1(a,b). However, the expectation values of the Kohn-Sham Hamiltonian for HOMO-1 and HOMO, which can be approximately interpreted as energy levels, alternated after 6 fs, which was consistent with the orbital character of the S_1_ state obtained by ΔSCF. This indicates that the change in the level ordering of the MO at particular atomic coordinates can automatically be searched by the rtp-TDDFT-MD simulation. The automatic on-the-fly identification of PES-crossing is an important advantage of the rtp-TDDFT-MD simulation.

**Figure 3:**
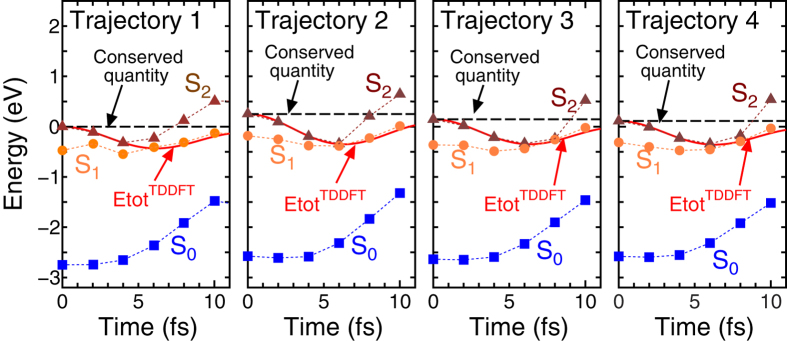
Comparison of the TDDFT potential energy and *static* DFT potential energies of S_n_ states with *n* = 0,1, and 2 for four trajectories of the rtp-TDDFT-MD simulation. Hatched triangles, circles, and squares are the total energies of the S_2_, S_1_, and S_0_ states, respectively, obtained by *static* DFT with common atomic coordinates in the snapshot of the rtp-TDDFT-MD simulations. The origin of the potential is set to that of the S_2_ state at beginning of “Trajectory 1”.

Finally in this subsection, we show that Fig. 3 indicates that the value of the potential energy obtained by the rtp-TDDFT-MD simulation does not show the intermediate value between those of the S_1_ and S_2_ states. Thus, we conclude that the mean-field average of PESs is not the case in the rtp-TDDFT-MD simulation of S_2_ excited azobenzene. The slight deviation in the values of the potentials in Fig. 3 may arise from a small amount of mixing between the S_1_ and S_0_ states, which is negligible considering the photoisomerization dynamics presented in the next subsection.

### Long-time simulation of photoisomerization

**Figure 4:**
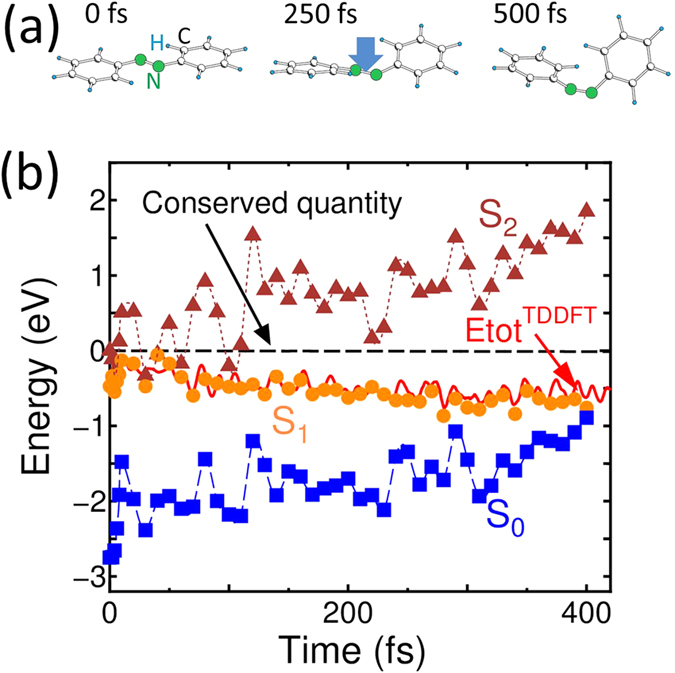
(**a**) Conformations of an azobenzene molecule at *t* = 0, 250, and 500 fs. Large green circles, small blue hatched circles, and open circles denote nitrogen, hydrogen, and carbon atoms, respectively. The vertical blue arrow denotes the initial motion of the N = N axis being pushed down out of the molecular plane. (**b**) Potential energy calculated by rtp-TDDFT-MD (solid red line) throughout the *trans-cis* conversion of an azobenzene molecule upon S_2_ excitation. Hatched triangles, circles, and squares denote *static* DFT total energies of S_2_, S_1_, and S_0_ states, respectively. The origin of the potential is set as that of the S_2_ state at *t* = 0 fs. The dashed line denotes the conserved quantity, and the potential energy plus kinetic energies of all ions.

The fact that rtp-TDDFT-MD beyond 6 fs follows a pure S_1_ state can also be demonstrated by a long-time simulation of the *trans-cis* photoisomerization of azobenzene. Because this long-time simulation is computationally expensive, we have chosen only “Trajectory 1” in Fig. 3 as a sample case. Figure 4(a) shows a conformational change in azobenzene in the long-time simulation. Up to 250 fs, the N = N axis started to move away from the molecular plane in the direction denoted by the blue arrow, and then at 250 fs two benzene rings began to lift from the original molecular plane. This is the beginning of the rotation pathway toward *cis*-azobenzene, which was achieved at *t* ~ 500 fs in the simulation. This time constant is the same order of magnitude as the constant obtained by the time-variation of the fluorescence data^33^,^34^, the energy-broadening factors of which indicate a lifetime of 200–700 fs. Statistical treatment of many trajectories of electron-ion dynamics including energy dissipation processes would be necessary for precise comparison, which is beyond the scope of the current work. We confirmed the (meta)stability of the last geometry of *cis*-azobenzene by performing conjugate-gradient geometry optimization in the S_0_ state (electronic ground state). The computed total energy of the *cis*-azobenzene is 0.8 eV higher than *trans*-azobenzene in the S_1_ state within the LDA level of accuracy.

We also compared our current results with recent simulations of azobenzene^35^,^36^,^37^,^38^,^39^. Non-adiabatic transitions using surface hopping technique within fewest-switch scheme were done by Ref. 35,39, while others^36^,^37^,^38^ studied profiles of PESs of excited states. Grannuci *et al.*^35^ reported a short time constant for both the S_2_ → S_1_ transition and the subsequent rotation pathway of the photo-isomerization within sub ps, which is consistent with our current results for photo-isomerization despite the different theoretical approach in ref. 35, *i.e.*, a combination of CASSCF and surface hopping with fewest switching mode. Meantime, the time constant around 6 fs for S_2_ → S_1_ transition in current simulation cannot be directly compared with ref. 35 because of the fewest-switch scheme. Other theoretical works^36^,^37^ using CASSCF^19^ formalisms and B3LYP^16^,^17^,^40^ functionals, showed the absence of a reaction barrier along with the rotational pathway and presence of the barrier along with inversion pathways upon S_1_ excitation. The current on-the-fly simulation starting from S_2_ excitation through relaxation to S_1_ excitation also shows no barrier for the rotational pathway, as seen in the potential-time plots in Figs 3 and 4(b). However, inversion pathways for photoisomerization that contradict our results have also been reported^38^,^39^. Theoretical conclusions about the pathway of photoisomerization are still under debate. However, this problem does not affect our current conclusion concerning the conservation of pure adiabatic states in the Ehrenfest dynamics because the potential mixing should be concerned only at a conformation region of the “cis”-azobenzene.

**Figure 5:**
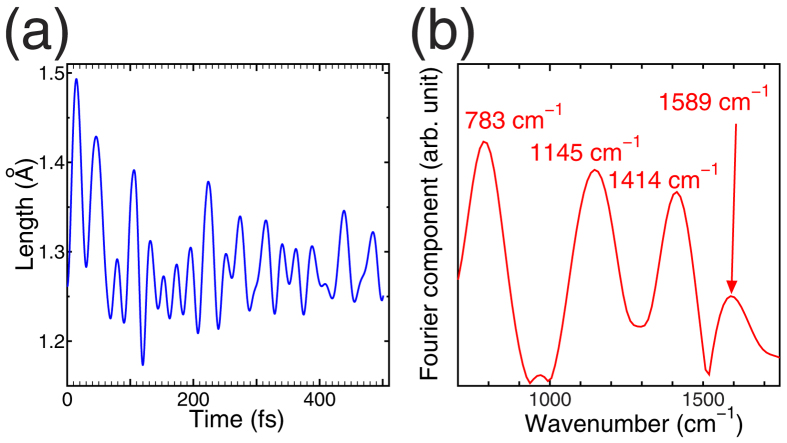
(**a**) Time-evolution of the N = N length of the azobenzene molecule throughout the dynamics displayed in Fig. 4. (**b**) Period domain of N = N stretching after the data in Fig. 5 (**a**) has been Fourier transformed. The corresponding wavenumbers expressing the frequencies are also displayed.

The oscillating behaviour in the potential in Fig. 4(b) arises from the induction of many vibration modes in the azobenzene molecule, such as bond stretching and bending, throughout the *trans-cis* conversion. During the isomerization, we monitored the N = N bond length as a function of time. Figure 5(a) shows the computed value and Fig. 5(b) shows its Fourier transformation in wavenumber domains. The Fourier transformation of the time-evolution of the N = N length shows two major peaks at 1414 and 1145 cm^−1^, which are consistent with the Raman spectra of photoexcited azobenzene at 1428 and 1130 cm^−1^^34^ within the possible error caused by LDA. These peaks are also consistent with recent theoretical work with higher accuracies in many-body treatments^32^,^41^ indicating the preservation of the double bond character of the N = N axis.

The potential energy obtained by the rtp-TDDFT-MD simulation in Fig. 4(b) was compared with those obtained by *static* DFT calculations of S_2_, S_1_, and S_0_ states along with the MD-trajectory. The long-time simulation showed that the potential of the rtp-TDDFT-MD simulation follows that of the S_1_ state instead of showing the mean-field average of PESs, and the potential goes near the S_0_ state after 400 fs.

### Results for the C_2_H_4_ molecule

The simulation in Fig. 4(b) could demonstrate the S_1_ → S_0_ transition or mean-field average of these states. However, examining azobenzene is too time-consuming because it is expected that the time-constant for the intersections of PESs of the S_1_ and S_0_ states will be beyond 500 fs at all MD-trajectories. We chose another common molecule, C_2_H_4_, which has *π* and π* orbitals equivalent to a 90° twist of the two CH_2_ components of the C_2_H_4_ molecule, which is similar to azobenzene photoisomerization.

**Figure 6:**
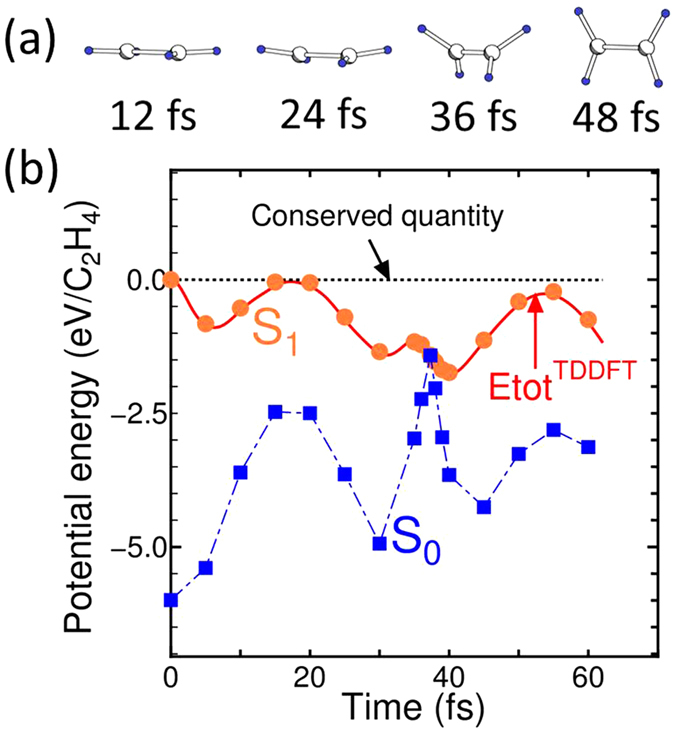
(**a**) Dynamics of a C_2_H_4_ molecule upon S_1_ excitation under high geometric symmetry conditions. (**b**) Corresponding potentials of the rtp-TDDFT-MD simulation and *static* DFT for the S_1_ and S_0_ states. Hatched squares and circles denote *static* DFT total energies of the S_0_ and S_1_ states, respectively.

Figure 6(a) shows the dynamics of the C_2_H_4_ molecules under S_1_ excitation. The initial atomic configuration was slightly twisted from the optimized structure under a periodic 10 × 5 × 5 Å^3^ box. The coordinates are listed in Table S5 in the supplementary material. There are three 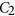 axes; one of the three is along the C-C bond axis and the other two are perpendicular to the C-C axis. The three 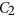 axes remain, even during photoisomerization of a C_2_H_4_ molecule keeping a constraint under the 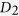 symmetry. Assuming no initial velocities on all atoms, the molecule immediately shows the rotation of the CH_2_ unit being accompanied by C-C stretching. Even within the LDA functional, the dynamics are consistent with quantum chemistry many-body wavefunction theory coupled with a surface-hopping technique^21^. Ben-Nun *et al.*^21^ also present further results for the intramolecular proton transfer derived from a lower symmetric configuration, which is beyond the scope of our work.

**Figure 7:**
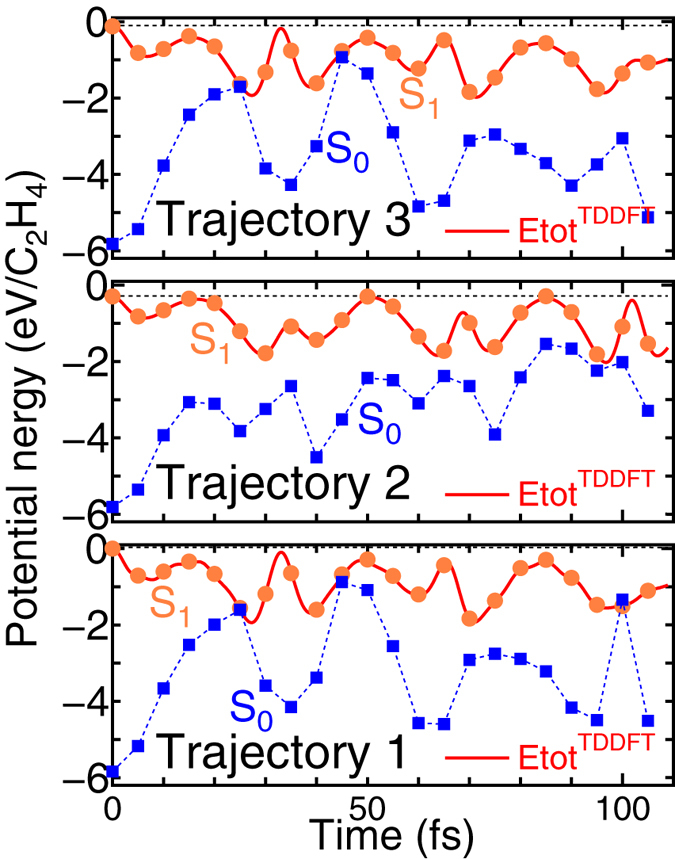
Potential energies on the three non-symmetric trajectories for the rtp-TDDFT-MD simulation of the S_1_ excited C_2_H_4_ molecule. Potential energies computed by *static* DFT for S_1_ and S_0_ states are also displayed. Hatched squares and circles denote *static* DFT total energies of the S_0_ and S_1_ states, respectively. The origin is set as the potential energy of the S_1_ state at the beginning of “Trajectory 1”.

Figure 6(b) shows a comparison of the potential energies obtained by ftp-TDDFT-MD and by *static* DFT for the S_1_ and S_0_ states. The simulation showed the intersection of PESs of the S_1_ and S_0_ states. Similar to the azobenzene simulation, the mean-field average of the PESs did not occur. The potential energy of rtp-TDDFT-MD coincided with the PES of the S_1_ state, and thus the S_1_ → S_0_ transition was inhibited. Considering the orbital occupation, the S_0_ state does not have a half-occupied MO, so a smooth transfer maintaining the numbers of fully and half-occupied MOs did not occur. We also examined the inhibition of the S_1_ → S_0_ transition and the mean-field average in the C_2_H_4_ molecule with no symmetric restriction on three MD trajectories. For the trajectories, see Tables S6 to S8 in the supplementary information, which show the initial conditions in the atomic coordinates and velocities of ions randomly distributed with a corresponding temperature of about 110 K. Here, the calculations were performed with a 10×10×10Å^3^ periodic box. The results are displayed in Fig. 7, where the intersection of S_1_ and S_0_ can be seen several times in all trajectories.

If the spin-polarized Kohn-Sham wavefunction is available, the S_1_ state can be expressed with a singly occupied LUMO and HOMO with up and down spins, respectively. If the time-evolution of the spin-up LUMO smoothly changes the orbital into a spin-up HOMO when the PESs of the S_1_ and S_0_ states intersect, the smooth S_1_ → S_0_ transition can be simulated. However, this is still not trivial in the rtp-TDDFT-MD scheme because there can also be an opposite change in wavefunction, namely changing the spin-down HOMO into a spin-down LUMO. If this occurs, we re-create an S_1_ state with the opposite spin configuration. Therefore, we expect that a smooth S_1_ → S_0_ transition is not guaranteed with the spin-polarized rtp-TDDFT-MD approach.

### Concluding remarks

The absence of the mean-field average of PESs may only occur in cases allowing neither charge transfer nor molecular disintegration. When disintegration occurs in the rtp-TDDFT-MD simulation, the numbers of distributed electrons are likely to be fractional. In this case, the simulation should be under a mean-field average of many PESs, in contrast to our present results. This case was also discussed in ref. 2.

However, the presence of many MOs may also explain the absence of the mean-field average particularly in current cases of rtp-TDDFT-MD. A C_2_H_4_ molecule has seven occupied MOs per spin for the S_1_ excited state within ΔSCF, whereas an azobenzene molecule has 35 occupied MOs per spin for S_2_ excited states. This number of MOs can interfere with each other through the Hartree-exchange-correlation potential of DFT during the rtp-TDDFT-MD simulation. This contradicts the two PES systems with a coherent Rabi-oscillation expressing the mean-field average of PESs. The interference becomes substantial as the number of wavefunctions increases and can suppress the mean-field average of PESs, setting the probability weighted on a particular PES. Interference among many orbitals is also seen in extended systems, similar to the relaxation of hot carriers in carbon nanotubes^42^.

In summary, we have demonstrated the absence of the mean-field average of PESs throughout the rtp-TDDFT-MD simulations for photoexcited azobenzene and C_2_H_4_ molecules. The S_2_ → S_1_ transition in azobenzene was observed in several trajectories followed by the *trans-cis* isomerization, which was consistent with previous experimental and theoretical studies. Conversely, the S_1_ → S_0_ transition was not observed in an S_1_ excited C_2_H_4_ molecule owing to the restriction of occupation numbers. Therefore, we concluded that each MD trajectory presented here was on a single PES and that the rtp-TDDFT-MD approach, at least for the current cases, is an on-the-fly tool for automatically monitoring transitions among PESs, unless the simulation reaches the PES of the S_0_ state. Further investigation of other molecules will help to generalize our findings.

## Additional Information

**How to cite this article**: Miyamoto, Y. *et al.* Conservation of the pure adiabatic state in Ehrenfest dynamics of the photoisomerization of molecules. *Sci. Rep.* **5**, 18220; doi: 10.1038/srep18220 (2015).

## Supplementary Material

Supplementary Information
