# Supplementary material for: Conservation of the pure adiabatic state in Ehrenfest dynamics of the photoisomerization of molecules
Source: Sci Rep. 2015 Dec 11;5:18220. doi: 10.1038/srep18220 (PMC4676005; doi:10.1038/srep18220)
Supplement: Supplementary Information [file srep18220-s1.pdf]

Supplementary material for "Conservation of the pure adiabatic state in Ehrenfest dynamics of the photoisomerization of molecules"

Yoshiyuki Miyamoto,[1] Yoshitaka Tateyama,[2] Norihisa Oyama,[3] and Takahisa Ohno[3]

[1]*Nanosystem Research Institute, National Institute of Advanced Industrial Science and Technology (AIST), Central 2, 1-1-1 Umezono, Tsukuba, Ibaraki 305-8568, Japan*

[2]*International Center for Materials Nanoarchitectonics (MANA), National Institute for Materials Science (NIMS), 1-1 Namiki, Tsukuba, Ibaraki 305-0044, Japan*

[3]*Computational Material Science Unit, Advanced Key Technologies Division, National Institute for Materials Science (NIMS), 1-2-1 Sengen, Tsukuba, Ibaraki 305-0047, Japan*

(Dated: November 3, 2015)

This supplementary material provides numerical data for atomic coordinates for azobenzene and atomic coordinates and velocities for ethylene (C_2H_4) molecules used as an initial condition of the rtp-TDDFT-MD simulations. All units are under atomic unit in Cartesian coordinates. Note that for trajectories displayed in tables S.1 to S.4, initial velocities were set as zero.

TABLE S.1: Initial atomic coordinates of an azobenzene molecule for trajectory 1, see main text.

atom	x(bohr)	y(bohr)	z(bohr)
C_1	3.2542339	3.4665378	0.0134931
C_2	5.5409594	4.7245627	0.0049001
C_3	7.8034388	3.3697666	0.0351071
C_4	7.7667243	0.7429504	0.0212516
C_5	5.4747315	-0.5297545	0.0166712
C_6	3.2169192	0.8288798	0.0450797
C_7	-3.2989255	-3.4233837	-0.0477802
C_8	-5.5861149	-4.6786283	-0.0063561
C_9	-7.8426260	-3.3265258	0.0543650
C_{10}	-7.8106797	-0.7040514	0.0337054
C_{11}	-5.5194183	0.5628479	0.0607014
C_{12}	-3.2630895	-0.7897739	0.0387064
H_1	1.4571594	4.4691617	-0.0436332
H_2	5.5818968	6.7816262	-0.0568690
H_3	9.6060152	4.3660070	0.0584859
H_4	9.5346589	-0.3091918	-0.0300775
H_5	5.3819464	-2.5855821	-0.0318740
H_6	-1.5035726	-4.4240632	-0.1184445
H_7	-5.6375098	-6.7417714	-0.0131674
H_8	-9.6373490	-4.3420949	0.1105494
H_9	-9.5769873	0.3521513	0.0261230
H_{10}	-5.4288933	2.6174854	0.1123012
N_1	0.9830349	-0.6115830	0.0523060
N_2	-1.0319837	0.6595248	0.0965901

TABLE S.2: Initial atomic coordinates of an azobenzene molecule for trajectory 2, see main text.

atom	x(bohr)	y(bohr)	z(bohr)
C_1	3.3389655	3.4763635	-0.0686640
C_2	5.6037426	4.6805904	-0.1794642
C_3	7.8925665	3.2999253	0.0031330
C_4	7.8092718	0.7198645	-0.0258110
C_5	5.4959272	-0.5654385	-0.0231130
C_6	3.2184931	0.8172669	0.0369061
C_7	-3.3454320	-3.4676046	-0.0128226
C_8	-5.6458731	-4.7055797	0.0357341
C_9	-7.8643618	-3.3123867	0.0096130
C_{10}	-7.7721306	-0.6833018	-0.0081002
C_{11}	-5.4779303	0.5855389	0.1426479
C_{12}	-3.2286658	-0.8195059	0.0531845
H_1	1.4967744	4.4126432	-0.1211853
H_2	5.6630879	6.7419417	-0.3771367
H_3	9.6964553	4.2708683	0.0446013
H_4	9.5237888	-0.4088625	0.0136737
H_5	5.3099294	-2.6352373	0.0167162
H_6	-1.5470172	-4.4944564	-0.0654469
H_7	-5.6270368	-6.7651292	0.0180151
H_8	-9.7138498	-4.2328570	-0.1177108
H_9	-9.4654660	0.4724722	-0.1108403
H_{10}	-5.3508033	2.6462563	0.2313078
N_1	0.9701062	-0.6450478	0.1213919
N_2	-0.9892613	0.6196340	-0.0694604

TABLE S.3: Initial atomic coordinates of an azobenzene molecule for trajectory 3, see main text.

atom	x(bohr)	y(bohr)	z(bohr)
C_1	3.3232849	3.4199459	-0.2020110
C_2	5.6463268	4.6287402	-0.2562948
C_3	7.8662861	3.2290449	-0.1396757
C_4	7.7640461	0.5892527	0.0099875
C_5	5.4975035	-0.6077104	0.0227082
C_6	3.2282506	0.8116901	-0.0007142
C_7	-3.3218563	-3.4547344	0.0249465
C_8	-5.6764918	-4.6048228	0.0352165
C_9	-7.8817651	-3.1837821	-0.0291295
C_{10}	-7.7904021	-0.5850518	0.0913607
C_{11}	-5.4587292	0.5965879	0.1644591
C_{12}	-3.2466048	-0.8292430	0.0836753
H_1	1.5981143	4.5286923	-0.2231602
H_2	5.7909514	6.6768917	-0.3235043
H_3	9.6657919	4.2492716	-0.1063054
H_4	9.4852918	-0.5491925	0.0364694
H_5	5.3154982	-2.6575066	0.0360737
H_6	-1.5337419	-4.4722116	-0.1090684
H_7	-5.8925079	-6.6575036	-0.0002189
H_8	-9.6904981	-4.1555571	-0.0328560
H_9	-9.5530440	0.4911843	0.1252082
H_{10}	-5.1899591	2.6387234	0.1991376
N_1	1.0283718	-0.6193434	0.2363495
N_2	-0.9842130	0.6013902	-0.0533344

TABLE S.4: Initial atomic coordinates of an azobenzene molecule for trajectory 4, see main text.

atom	x(bohr)	y(bohr)	z(bohr)
C_1	3.3386223	3.3507974	-0.2719016
C_2	5.6767941	4.5549954	-0.3739621
C_3	7.8973681	3.1272059	-0.2893551
C_4	7.7664814	0.4985402	-0.0581534
C_5	5.5005355	-0.6755429	0.1823342
C_6	3.2294316	0.7560596	-0.0361139
C_7	-3.3682607	-3.4043937	0.1253740
C_8	-5.7127155	-4.5620169	-0.0841233
C_9	-7.9420138	-3.1020524	0.0642828
C_{10}	-7.7583543	-0.4730075	0.2180076
C_{11}	-5.4225065	0.6976461	0.1470097
C_{12}	-3.2298669	-0.7549470	0.0883018
H_1	1.5986263	4.4048386	-0.5134232
H_2	5.8180712	6.6028933	-0.4080231
H_3	9.7448087	4.0557209	-0.4432724
H_4	9.5342805	-0.5470580	0.1267586
H_5	5.2146396	-2.6891332	0.4864187
H_6	-1.6458185	-4.5167771	-0.0399363
H_7	-5.8021804	-6.6306412	-0.1051614
H_8	-9.7805523	-4.0096608	-0.0067661
H_9	-9.4484567	0.7177836	0.3438617
H_{10}	-5.2052249	2.7437262	0.0301011
N_1	1.0024203	-0.6535861	0.2464727
N_2	-0.9825662	0.6298811	0.0254533

TABLE S.5: Initial atomic coordinates x, y, z and velocity $v_x, v_y, v_z$of an ethylene molecule for the symmetric trajectory under D_{2d} symmetry, see main text.

atom	x(bohr)	y(bohr)	z(bohr)	v_x(a.u.)	v_y(a.u.)	$v_z(a.u.)$
C_1	1.2475986	0.0000000	0.0000000	0.0	0.0	0.0
C_2	-1.2475986	0.0000000	0.0000000	0.0	0.0	0.0
H_1	2.3204950	1.7514560	0.0108269	0.0	0.0	0.0
H_2	2.3204950	-1.7514560	-0.0108269	0.0	0.0	0.0
H_3	-2.3204950	1.7514560	-0.0108269	0.0	0.0	0.0
H_4	-2.3204950	-1.7514560	0.0108269	0.0	0.0	0.0

TABLE S.6: Initial atomic coordinates x, y, z and velocity $v_x, v_y, v_z$of an ethylene molecule for the nonsymmetric trajectory 1, see main text.

atom	x(bohr)	y(bohr)	z(bohr)	v_x(a.u.)	v_y(a.u.)	$v_z(a.u.)$
C_1	1.2531151	-0.0953752	0.0321567	0.0000346494143653	-0.0000695829521473	0.0000104416900752
C_2	-1.2518629	0.0997111	-0.0317653	-0.0000262428052399	0.0000796465942467	-0.0000118897013747
H_1	2.5475107	1.5728268	-0.2470178	-0.0001779746127869	0.0002444175196515	-0.0004213359676168
H_2	2.1076585	-2.0274099	0.3515273	-0.0004069967125997	-0.0003480390311969	0.0005165788666921
H_3	-2.1947888	1.9714273	-0.3096237	0.0002872986234283	0.0002015428153979	0.0000962257151826
H_4	-2.4756769	-1.5691163	0.2001749	0.0001965268566443	-0.0002189110061531	-0.0001746184999408

TABLE S.7: Initial atomic coordinates x, y, z and velocity $v_x, v_y, v_z$of an ethylene molecule for the nonsymmetric trajectory 2, see main text.

atom	x(bohr)	y(bohr)	z(bohr)	v_x(a.u.)	v_y(a.u.)	$v_z(a.u.)$
C_1	1.2671807	-0.2037534	0.0582555	0.0000523439375806	-0.0000449584649413	-0.0000052345857261
C_2	-1.2652565	0.2103543	-0.0591575	-0.0000258306262199	0.0000220700601932	0.0000054380620377
H_1	2.6053730	1.2123708	-0.4152956	0.0000549049869983	-0.0000372426711391	0.0000975193770347
H_2	2.0284930	-1.9577665	0.6352456	-0.0002524558091713	-0.0001772923030347	0.0000904466977309
H_3	-2.1005971	1.9940188	-0.5357136	-0.0000414197703221	0.0002610774823571	-0.0004206091375505
H_4	-2.5569919	-1.3280730	0.3254079	-0.0000798100973733	0.0002282606622928	0.0002294929605233

TABLE S.8: Initial atomic coordinates x, y, z and velocity $v_x, v_y, v_z$of an ethylene molecule for the nonsymmetric trajectory 3, see main text.

atom	x(bohr)	y(bohr)	z(bohr)	v_x(a.u.)	v_y(a.u.)	$v_z(a.u.)$
C_1	1.2642361	-0.1201792	0.0348592	-0.0000585089842922	-0.0000993006020677	0.0000079671578706
C_2	-1.2584666	0.1254288	-0.0351611	0.0000432140708706	0.0000735945128540	-0.0000087807406013
H_1	2.4355240	1.4673814	-0.2924290	-0.0000248263071977	-0.0002874238784862	-0.0000468910126038
H_2	2.0454000	-1.9156400	0.4245652	0.0003410972087300	0.0002391789033325	0.0001958735179792
H_3	-2.1257143	1.9271613	-0.3003397	-0.0000969375260580	0.0001163016407763	-0.0001608308134016
H_4	-2.4248056	-1.5421465	0.1714533	-0.0000359320615857	0.0002406490129808	0.0000211904744063
